# Exploring clade differentiation of the *Faecalibacterium prausnitzii* complex

**DOI:** 10.1016/j.isci.2022.105533

**Published:** 2022-11-09

**Authors:** Marco Fabbrini, Marco Candela, Silvia Turroni, Patrizia Brigidi, Simone Rampelli

**Affiliations:** 1 Microbiomics Unit, Department of Medical and Surgical Sciences, University of Bologna, Bologna 40138, Italy; 2 Unit of Microbiome Science and Biotechnology, Department of Pharmacy and Biotechnology, University of Bologna, Bologna 40126, Italy

**Keywords:** Bacteriology, Microbial genomics, Microbiome

## Abstract

*Faecalibacterium prausnitzii* is one of the most prevalent and abundant polyphyletic health-promoting components of the human gut microbiome with a propensity for dysbiotic decreases. To better understand its biology in the human gut, we specifically explored the divergence pressures acting on *F. prausnitzii* clades on a global scale. Five *F. prausnitzii* clades were *de novo* identified from 55 publicly available genomes and 92 high-quality metagenome assembled genomes. Divergence rate indices were constructed and validated to compare the divergence rates among the different clades and between each of the diverging genes. For each clade we identified specific patterns of diverging functionalities, probably reflecting different ecological propensities, in term of inter-host dispersion capacity or exploitation of different substrates in the gut environment. Finally, we speculate that these differences may explain, at least in part, the observed differences in the overall divergence rates of *F. prausnitzii* clades in human populations.

## Highlights

• Five *Faecalibacterium prausnitzii* clades were *de novo* identified
• Divergence rate indices were developed at clade level and gene level
• Specific patterns of diverging genes were identified for each clade
• Patterns concern inter-host dispersion and a better exploitation of the gut environment

## Graphical abstract

**Figure:**
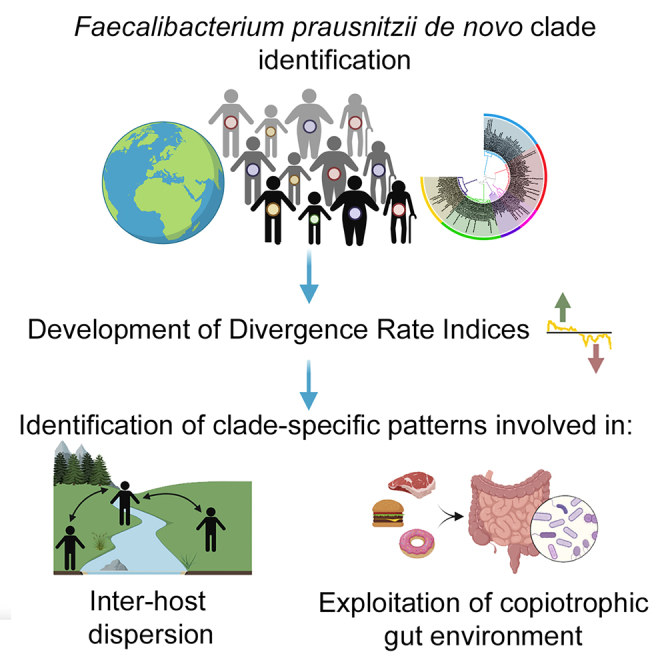

## Introduction

*Faecalibacterium prausnitzii* is one of the most wide-spread and abundant bacteria in the human gut microbiome (GM). It is probably an integral component of our evolutionary history which has populated our lineage for at least 1M years.^1^ *F. prausnitzii* has been consistently reported as one of the main health-promoting components found in the intestine,^2^ showing a crucial role in host nutrition and immunity, where it acts as an important anti-inflammatory commensal.^3^ Indeed, recent studies^4^^,^^5^^,^^6^ have shown that *F. prausnitzii* can attenuate the severity of inflammation through the release of a panel of anti-inflammatory metabolites, which enhance the intestinal barrier acting on tight junctions, as well as on peroxisome proliferator-activated receptor alpha (PPAR-α), PPAR-γ and PPAR β/δ genes.^7^

Over the last few years an increasing number of studies have reported a depletion of *F. prausnitzii* in GMs associated with multiple diseases, enteric and non-enteric,^8^^,^^9^^,^^10^^,^^11^^,^^12^ to the point that this bacterium has been proposed as a possible biomarker of dysbiotic shifts. This defines a complex scenario where, on the one hand, *F. prausnitzii* has a crucial role in maintaining gut homeostasis, but on the other hand it is extremely prone to dysbiotic reductions. However, at present, it still remains elusive which biotic and abiotic factors regulate its presence in the gut, the extent of their influence and the mechanisms involved in its retention.

First *16S rRNA* gene-based phylogenetic analyses showed that at least two different *F. prausnitzii* phylogroups can be found in the human GM, whose distribution is different between healthy subjects and patients with gut disorders.^13^^,^^14^ Most recently, the polytypic nature of *F. prausnitzii* has been confirmed, detecting up to 11 different clades, which show a different prevalence and/or abundance in the human GM depending on age, geographical origin and lifestyle.^15^ These authors also confirmed the depletion of this species in inflammatory bowel disease and obesity. Although these findings certainly represent a milestone for a better understanding of *F. prausnitzii* biology in the human gut, there is still no evidence concerning possible selective pressures driving for the observed clades divergences, and it has not yet been investigated why such clades exhibited a markedly different distribution in the human population.

In an attempt to answer these questions, here we explored the dynamics involved in the divergence processes of the clade-specific marker genes in the *F. prausnitzii* complex, dissecting the peculiarities of each clade and providing some glimpses on the putative pressures selectively acting on each of them. Specifically, we reconstructed high-quality *F. prausnitzii* genomes from metagenomes (MAGs) starting from ∼750 healthy human gut metagenomes^16^^,^^17^^,^^18^^,^^19^^,^^20^^,^^21^^,^^22^ and identified *F. prausnitzii* clades by implementing a previously validated pipeline.^15^^,^^23^^,^^24^ Then, the within-clade genetic diversity have been analyzed, allowing to dissect the putative evolutionary forces acting on each clade. In particular, the divergence dynamics were assessed by accounting for the specific pattern of mutations accumulating in the respective clade-specific genes. Given the high susceptibility of this species to alteration of host homeostasis and environmental stresses, our findings may provide new insights into the determinants responsible for its decrease in disease conditions and help to find solutions for the recovery of this keystone taxon.

## Results

### *De novo* identification and functional characterization of 5 *F. prausnitzii* clades

**Figure 1:**
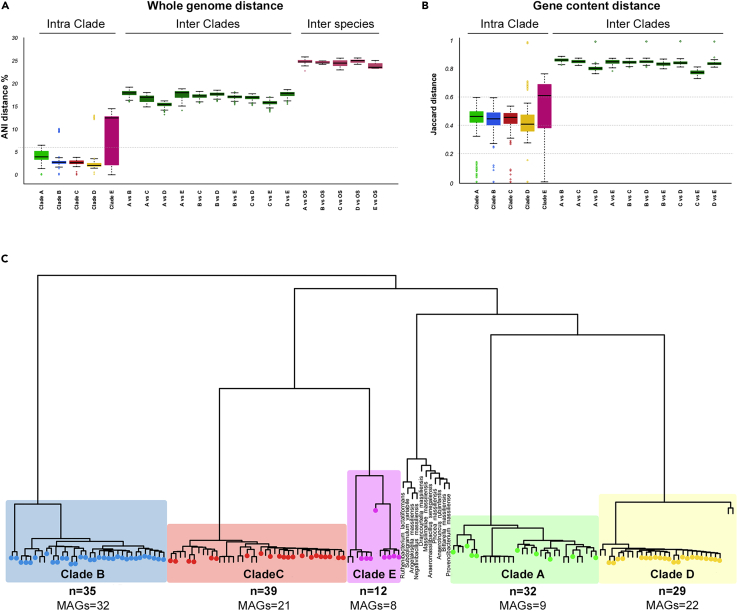
*De novo* identification and functional characterization of 5 *Faecalibacterium prausnitzii* clades (A) Genetic distances in terms of ANI, within a clade (intra-clade), between clades (inter-clade) and between clades and other species (OS) of the Ruminococcaceae family, used as outgroups (see STAR Methods). Five *F. prausnitzii* clades (A to E) were identified. The dotted line denotes the 6% ANI distance threshold. (B) Jaccard distance based on gene content between (inter-clade) and within (intra-clade) *F. prausnitzii* clades. (C) Whole-genome phylogenetic tree derived from PhyloPhlAn2, representing the genome panel (n = 158, of which 92 MAGs, 55 reference genomes and 11 OS) clustered into the 5 identified clades. Colored circles indicate the genomes we assembled from metagenomes (MAGs).

We assembled 92 high-quality *F. prausnitzii* MAGs from 740 human gut metagenomes from a corresponding number of healthy subjects from 7 different studies, representing 8 different populations (Germans, Italians, Swedes, North Americans, Japanese, Peruvians and Tanzanian hunter-gatherers) (Figure S1). The obtained MAGs showed >95% completeness and <5% contamination levels.^25^ These 92 MAGs were complemented with 55 *F. prausnitzii* genomes directly downloaded from the NCBI RefSeq database (https://www.ncbi.nlm.nih.gov/refseq) (Table S1), for a total of 147 genomes used for the subsequent analyses. By computing the average nucleotide identity (ANI) distances, the Jaccard dissimilarity matrix on genes content and the PhyloPhlAn2^26^ phylogenetic grouping, we were able to identify 5 clades (A to E), with the largest (clade C) hosting 39 genomes and the smallest (clade E) comprising 12 genomes (Figure 1). By means of alignments, we noted that the 11 clades previously reported by DeFilippis et al.^15^ were represented within ours (Table S2). Arguably, the higher completeness threshold we applied for MAGs assembly explains the lower number of clades we were able to identify in our study. When we sought for functional specificities, we observed considerable functional differences between our clades, in terms of presence/absence of specific KEGG Orthology (KO) functionalities (Figure S2) and carbohydrate-active enzymes (CAZymes) (Figure S3). Most of the differences in KO genes concerned broad cellular processes, such as energy metabolism, ABC transporters and dehydrogenases. As regards carbohydrates metabolism, clade A was the most eclectic, bearing the highest fraction of CAZymes, followed by clades D and E. In contrast, clade B seemed to behave as a specialist, possessing a lower amount of CAZymes showing a particularly underrepresented glycoside hydrolase functional potential.

We next assessed the distribution of the 5 clades in the human population (see STAR Methods). According to our data, the 5 clades we identified are distributed across the entire set of human populations considered, thus all the clades can be regarded as cosmopolitan (Figure S4A). To investigate if these clades were mutually exclusive or able to co-inhabit the bowel, we evaluated the co-presence within the same metagenomic sample. This analysis clearly revealed that the degree of co-presence is variable in the human population, with some subjects harboring all the clades, whilst others not harboring *F. prausnitzii* at all. In particular, we observed that the presence/copresence of the *F. prausnitzii* clades was associated with age, geographical origin and subsistence strategy (Figures S4B–S4D), confirming what previously highlighted in another study (De Filippis et al., 2020^15^). Indeed, *F. prausnitzii* was almost always present in adults (96% contained at least 1 *F prausnitzii* clade, 18–69 years old), but the prevalence considerably decrease in infant (29%, <1 years old, Fisher’s test p<0.01), and centenarians (40%, >99 years old, p<0.01). Lower prevalence was also detected in children (89%, 1–16 years old, p<0.01) and elderly people (89%, 70–97 years old, p<0.01). Finally, the intra-individual clades diversity was highly variable according to the geographical area and the related lifestyle, with higher levels in non-Western countries (e.g., Tanzania, Wilcoxon test p=0.0001), respect to Western countries, that showed a progressively lower prevalence for all clades from Europe to Japan through North America.

### Construction and validation of divergence indices

To account for the rate of divergence between the *F. prausnitzii* clades, we developed two Divergence Rate Indices (DRIs), one at the clade level and the other at the gene level. The clade-level DRI (DRIc), was specifically conceived to account for the overall divergence rate of each clade and was computed as the natural logarithm (ln) of the ratio between the median number of single nucleotide polymorphisms (SNPs) of the whole set of clade-specific genes (M_E_) – defined as genes present in at least the 95% of the genomes from a given clade and absent in the other clades – and the median SNPs for a basal set of housekeeping reference genes (M_H_), *i.e.*, genes showing a little divergence within a clade. The housekeeping references was constituted by a panel of 10 genes (*recA*, *rplS*, *rplI*, *purN*, *mreB*, *maf*, *fmt*, *gyrB*, *rpoB*, *proC*) (Table S3), comprising essential genes we found present in all the genomes and MAGs we analyzed. On the other hand, the gene-level DRI (DRIg) was created to account for the absolute divergence rate for a single clade-specific gene for a given clade and was computed as the ln of the ratio between the SNPs of the selected clade-specific gene (M_G_) and M_H_ (the median SNPs of the basal set of housekeeping reference genes).

Further, to fully assess divergence pressures, the aforementioned indices were implemented by considering the ratio of non-synonymous to synonymous substitutions (dN/dS).^27^ Consistently, 2 non-synonymous divergence rate indices (NDRIs) were developed: (1) The clade-level NDRI (NDRIc), which considers the ln of the ratio between the mean dN/dS values for the whole set of clade-specific genes (μ_E_) and the mean dN/dS for the basal set of housekeeping reference genes (μ_H_), (2) the gene-level NDRI (NDRIg), as ln of the ratio between the dN/dS value of the selected clade-specific gene (μ_G_) and μ_H_.

Generally, for a given clade, a positive value for the DRIc and NDRIc indices points out that the corresponding set of clade-specific genes are accumulating SNPs and non-synonymous SNPs faster than housekeeping references; the higher the index values, the greater the divergence rate for the specific clade. Analogously, for a given clade-specific gene, a positive value for the DRIg and NDRIg indices indicates that the gene is accumulating SNPs and non-synonymous SNPs faster than housekeeping references; the higher the index values, the greater the divergence rate for the given clade-specific gene.

Because the DRIg and NDRIg indices were first necessarily computed at the level of the single metagenomes, to be then extrapolated at the population and metapopulation levels, and to verify any bias due to the sequencing yields, for each clade we performed Pearson’s correlation tests between M_H_ and μ_H_ values and metagenome lengths and the computed *F. prausnitzii* abundances. Correlations were also sought between gene prevalence and DRIg/NDRIg indices, to assess the presence of biases due to sequencing coverage on specific genes. According to our findings, no significant correlations were found (p>0.05).

### Divergence dynamics: Each clade shows a distinctive profile

**Figure 2:**
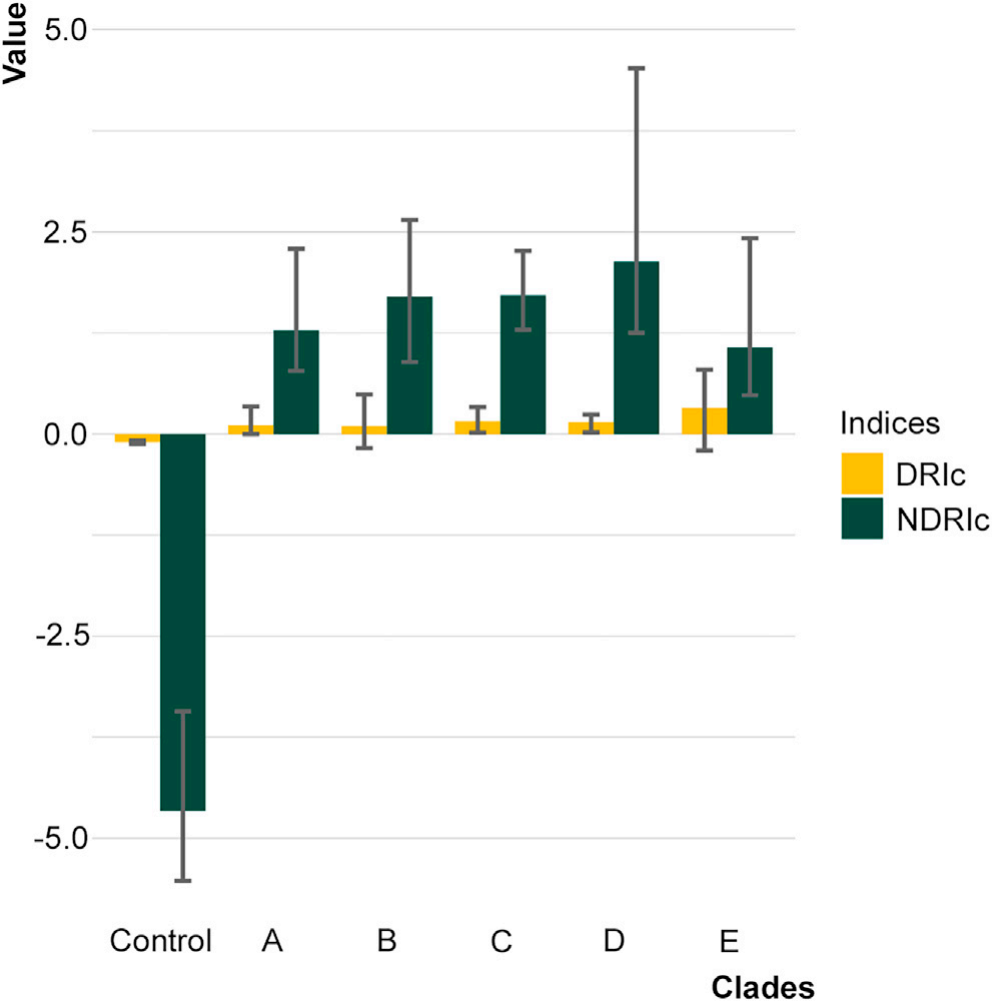
Clade-specific marker genes showed higher divergence indices than core genes For each metagenomic sample we computed the DRIc and NDRIc values for the 5 *F prausnitzii* clades detected (A to E). Median values among the 740 samples investigated are shown with whiskers ranging from the 25^th^ to the 75^th^ percentiles. Control refers to 500 core genes taken from the pan-genome of the *F. prausnitzii* complex as detected by the ROARY^28^ pipeline. For further information concerning DRI and NDRI calculation and marker-genes/core-gene identification, consult the STAR Methods.

Once defined and validated, we utilized our indices to study the divergence of the *F. prausnitzii* complex in the human population. First, we assessed the divergence of the clades in the human population by calculating the global DRIc and NDRIc indices (Figure 2) as the median of all the DRIc and NDRIc indices computed for the single metagenomic samples. For each clade, both global DRIc and NDRIc indices showed positive values, in contrast to the global indices for 500 randomly picked core genes (see STAR Methods), which resulted in negative values. This confirms that clade-specific marker genes are globally accountable for the divergence of the clades; hence, investigating their function may provide new glimpses over the selective pressure driving clades divergence. In particular, clade D showed the highest NDRIc values - with relatively high values for the DRIc index as well – resulting in the most rapidly diverging clade in the human population.

**Figure 3:**
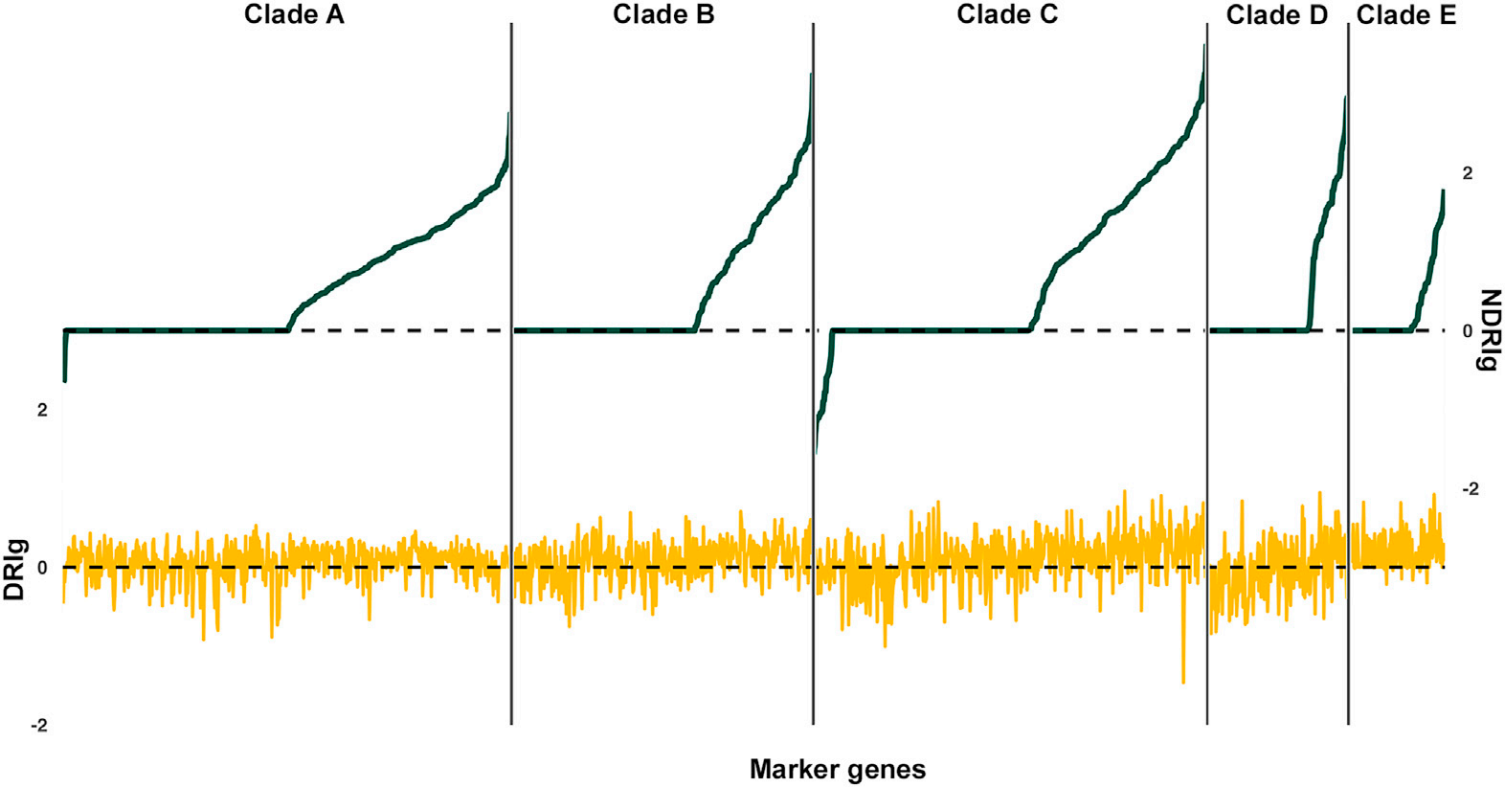
Clade-specific marker genes show different values of DRIg and NDRIg indices Curves represent the median values of the DRIg (yellow, bottom) and NDRIg (green, top) indices across the 740 metagenomic samples for each clade-specific marker gene. Genes are in increasing order with respect to the NDRIg values. See also Table S4 for the DRIg and NDRIg values for each clade-specific marker gene and Table S7 for the number of marker genes for each clade.

Next, to highlight for each clade the most diverging clade-specific genes, the clade-specific patterns of DRIg and NDRIg indices were computed (Figure 3 and Table S4). For each clade, gene-level divergence indices showed positive values for a multitude of clade-specific genes, indicating an overwhelming divergence rate in the human population that far exceeds that characteristic of housekeeping genes, as representative of a basal divergence.

For each clade, marker genes were then filtered, keeping only those with both global DRIg and NDRIg positive values. We interpreted the combination of higher mutation rates and more impactful mutations as a signature of active divergence of those regions, therefore investigating the function of such sequences may provide new glimpses over the selective pressures acting globally on *F. prausnitzii*.

### Clade-specific marker genes show genetic signatures of purifying selection and selective sweeps

**Figure 4:**
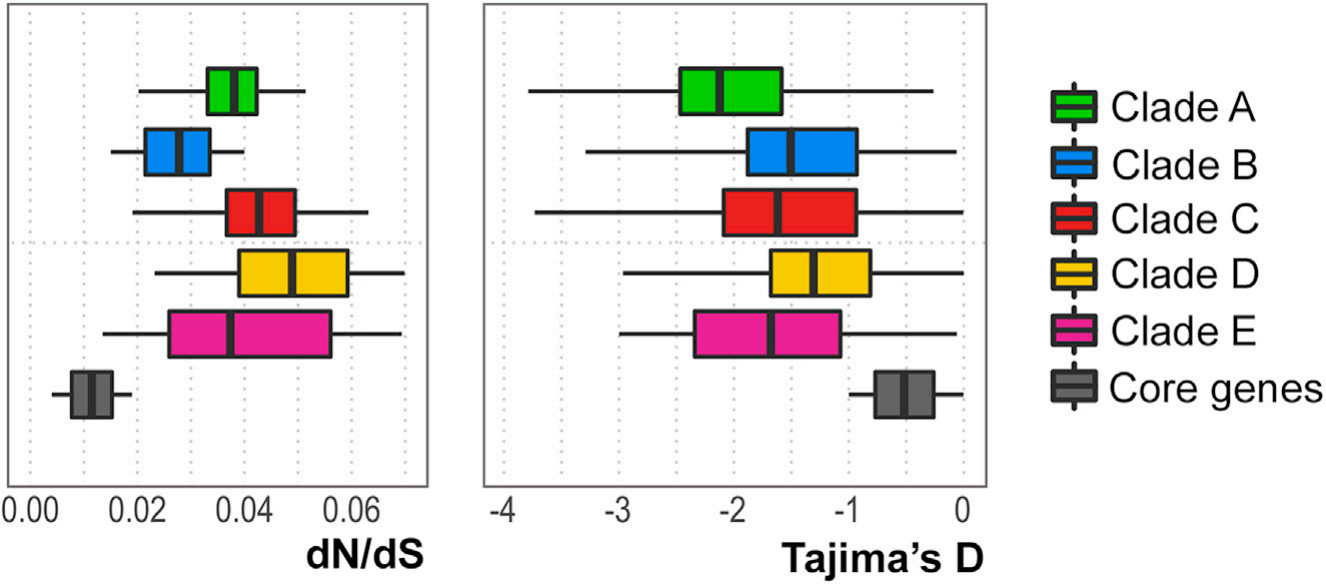
Clade-specific marker genes show genetic signatures of purifying selection and selective sweeps dN/dS and Tajima’s D estimates were computed on clade-specific marker genes and 500 core genes, the same defined in Figure 2. The marker genes showed higher level of purifying selection estimated through the ratio of non-synonymous to synonymous nucleotide substitutions (dN/dS) and Tajima’s D values respect to core genes.

To confirm that clade-specific marker genes are evolving under a non-neutral process, we added Tajima’s D^29^ to our approach. This parameter allows one to identify sequences that do not fit the neutral theory model at equilibrium between mutation and genetic drift. Computing Tajima’s D for *F. prausnitzii* on synonymous sites, to reduce the effects of selection, we observed negative values for all 5 clades (mean −1.6), with clade A showing the lowest value (−2.1) and clade D the highest (−1.3). Looking at the single gene contributions, we found that clade-specific marker genes contributed more to the negative values than core genes, indicating strong level of purifying selection with an excess of rare polymorphisms (Figure 4). Also, together with the evidence from our indices, these estimates suggest that the higher values of the dN/dS ratio of the marker genes are probably caused by recent mutations, capturing a current selection still in progress, acting immediately after or in a context of selective sweeps.

### Different clades show different functions of the clade-specific marker genes under divergence pressure

**Figure 5:**
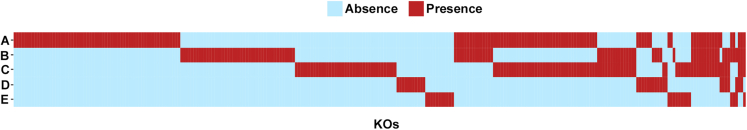
Different clades show different functions of the clade-specific marker genes under divergence pressure Five hundred and fifty-five clade-specific marker genes with NDRIg and DRIg >0 were classified in the KEGG database and visualized for their *F. prausnitzii* clade (A to E)-specific presence. Red, presence; cyan, absence. See also Table S5 for the complete list of KOs under divergence pressure for each clade.

To investigate the function of clade-specific marker genes filtered according to the combination of DRIg and NDRg indices, KEGG Orthology^30^ was used, allowing one to take into account the possible functional redundancy among the different markers. Thus, for each clade, we were able to obtain a profile of KOs corresponding to the most diverging clade-specific genes, *i.e.*, those showing positive DRIg and NDRIg values. As expected, several KOs were specific to each single clade, whilst others were shared by two to four clades. No common functions to all clades were identified (Figure 5 and Table S5).

In particular, clade A showed 64 distinctive KOs, including many genes related to sporulation, DNA repair, microbial resistance mechanisms (*e.g.*, antibiotic biosynthesis, xenobiotic degradation, CRISPR proteins, CAMP-resistance) and several transporters and transcription factors. As for clade B, we identified 43 unique KOs, mainly concerning the two-component system, antibiotic resistance genes, membrane transporters, as well as DNA repair and one carbon pool by folate. Clade C presented 39 selective KOs involved in DNA repair, sporulation, antimicrobial resistance, beta-lactam resistance, xenobiotic degradation, as well as several efflux proteins, transcription factors, genes involved in tRNA biogenesis, ribosome biogenesis and aminoacyl-tRNA biosynthesis. In addition to these functions, Clade C was the only clade that showed the anti-inflammatory MAM (microbiota anti-inflammatory molecule) protein within the filtered marker genes. Clade D and clade E exhibited 11 specific KOs, with the first particularly enriched in inorganic ion transporters and functions related to amino acids metabolism and transport, and the second in carbohydrate and lipid transporters (Table S6).

**Figure 6:**
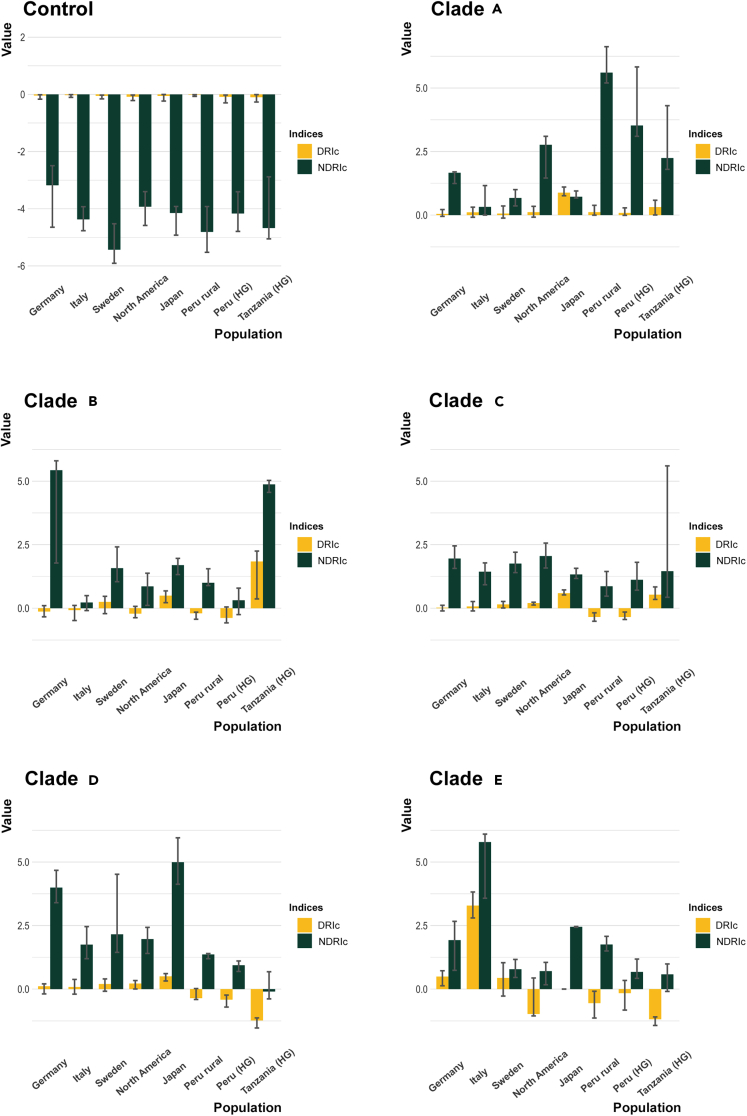
Clade-level indices show differences among the considered human populations DRIc and NDRIc indices were computed at the single population level as the median of the individual DRIc and NDRIc values among the subjects belonging to that population. For each clade (A–E), divergence rates showed sign of active divergence compared to the housekeeping Control. Clade C resulted particularly consistent across all populations, whilst clade A and D showed opposite trends, being respectively highly divergent in rural communities and industrial urban populations. The 25^th^ and 75^th^ percentiles are shown with whiskers. Control refers to the 500 core genes as in Figure 2. The following populations were considered: industrial urbans from Germany, Italy, Sweden, North America and Japan, rural inhabitants from Peru, and hunter-gatherers (HG) from Peru and Tanzania.

Finally, for each clade, we explored the variation in clade-level divergence rates in different human populations. According to our findings, all clades, with the exception of clade C, showed a heterogeneous pattern of DRIc and NDRIc in the human populations considered (Figure 6). In particular, quite opposite trends were found for clades A and D, with the former showing the highest divergence rates in hunter-gatherers and rural communities, and the latter diverging most actively in industrial urban populations.

## Discussion

Starting from previous evidences^12^^,^^14^^,^^15^ that *F. prausnitzii* is a polytypic species, we performed a *de novo* clade identification process and then took a step forward to gauge possible determinants of clades divergence. In particular, by analyzing a panel of 92 *F prausnitzii* MAGs assembled from 740 human metagenomes and 55 available genomes from NCBI, we were able to define 5 distinct clades of the *F. prausnitzii* complex, on which we based our further research. Four divergence rate indices (DRIc, NDRIc, DRIg and NDRIg) were constructed and validated, which, combined with Tajima’s D estimation, allowed for a curated assessment of the non-neutral divergence rate of each clade down to the gene level. Of interest, the exploitation of gene-level indices to identify the most rapidly diverging clade-specific marker genes allowed us to dissect the signatures of the possible selective pressures acting over these clades. In particular, for the clades A, B and C, the most rapidly diverging genes corresponded to functionalities that may allow to better cope with environmental changes, as well as to increase the inter-host dispersion capacity. Indeed, clade A was found to rapidly diverge in genes involved in several stages of the sporulation process, DNA repair and microbial resistance mechanisms, all of which are important factors for a prokaryotic cell to withstand and counteract environmental stresses. Similarly, clade B revealed a propensity to diverge functionalities related to the two-component system, mRNA expression regulation, aminoacyl-tRNA biosynthesis, transporters and membrane proteins, which may allow for a better metabolic flexibility in response to environmental stimuli. Finally, clade C combined a certain resistance potential, attributable to DNA repair, sporulation and resistance genes, with functional adaptability, as evidenced by several genes encoding transcription factors and transporters, and involved in the expression regulation. As a distinctive feature of clade C, the most rapidly diverging clade-specific genes also included functions related to the modulation of the immune response, such as the MAM protein, which has been shown to exert anti-inflammatory activities primarily via NF-κB pathway inibition.^31^ In contrast, clade D and E showed a different pattern of diverging functionalities, probably related to the exploitation of a copiotrophic gut environment, as indicated by the distinctive presence of genes coding for amino acid transport and metabolism, as well as carbohydrates and lipids transporters.

In our study, we found differences in the clade-level divergence rates between different human populations. In particular, clade A is diverging faster in hunter-gathering and rural populations, whereas clade D showed an opposite trend. Taken together, these observations might suggest that *F. prausnitzii* clades – or at least some of them - are evolving characteristic functional specializations that are better suited to the context of a specific host subsistence strategy which, in turn, would favor a more rapid divergence rate. For instance, clade A – which is evolving functionalities to survive outside host – showed a better fit in traditional populations, where inter-host dispersion of GM components is still an active process as it is not compromised by the sanitization practices typical of Western populations.^32^^,^^33^ Conversely, clade D, which is evolving adaptations for efficient exploitation of different substrates within the gut environment, showed a better fit and faster adaptive evolution in industrial urban populations, who are well known to consume high-fat/high-protein diets enriched in simple carbohydrates.^34^ Future studies including the isolation and cultivation of different *F. prausnitzii* strains representing each clade should be crucial to better identify the specific selective pressures driving clade differentiation.

Overall, our findings may provide new insights into the possible factors driving to the differentiation of the observed subspecies groups in the *F. prausnitzii* taxon. This information may be helpful for better understanding the evolutionary propensity of this health-promoting GM component allowing, from our side, to adopt sustainable dietary and lifestyle practices to favor its retention in the human gut. This is particularly important for industrial urban populations, where a decrease in *F. prausnitzii* diversity and prevalence has been observed.^15^ Possibly, the excess of sanitization practices typical of these populations is just facilitating the reduction of the *F. prausnitzii* clades A-C, which are evolving for better outside-host survival as a strategic factor for increasing their colonization of the human population.

Finally, the procedure we developed and implemented in this work can be virtually applied to every polytypic species of bacteria and, assuming the use of a sufficient number of genomes and metagenomes, could provide new ecological insights over the evolutionary forces shaping the microbial world around and within us.

## STAR★Methods

### Key resources table

**Table:** 

REAGENT or RESOURCE	SOURCE	IDENTIFIER
**Deposited data**		
*Faecalibacterium prausnitzii* reference genomes	NCBI	Accession numbers reported in Table S1
Human gut metagenomes	Asnicar et al.^16^	Accession numbers reported in Table S1
Human gut metagenomes	Backhed et al.^18^	Accession numbers reported in Table S1
Human gut metagenomes	Biagi et al.^19^	Accession numbers reported in Table S1
Human gut metagenomes	Costea et al.^17^	Accession numbers reported in Table S1
Human gut metagenomes	Nishijima et al.^20^	Accession numbers reported in Table S1
Human gut metagenomes	Obregon Tito et al.^21^	Accession numbers reported in Table S1
Human gut metagenomes	Rampelli et al.^22^	Accession numbers reported in Table S1
*Rumiococcaceae* reference genomes (here termed “Other Species - OS”)	NCBI	NCBI: PRJNA224116

**Software and algorithms**		
SRA toolkit 2.8.0	Leinonen, Sugawara and Shumway, 2011	https://github.com/ncbi/sra-tools
FastQC 0.11.8	Andrews,^35^	http://www.bioinformatics.babraham.ac.uk/projects/fastqc
KneadData 0.7.2	McIver et al.^36^	https://github.com/biobakery/kneaddata
MetaWRAP 1.0.2	Uritskiy et al.^37^	https://github.com/bxlab/metaWRAP
MetaPhlAn2 2.7.5	Truong et al.^38^	https://github.com/biobakery/MetaPhlAn
MegaHIT 1.1.2	Li et al.^39^	https://github.com/voutcn/megahit
MetaBAT2 2.12.1	Kang et al.^40^	https://bitbucket.org/berkeleylab/metabat
MaxBin2 2.2.5	Wu et al.^41^	https://sourceforge.net/projects/maxbin2/
CheckM 1.0.7	Parks et al.^42^	https://github.com/Ecogenomics/CheckM/wiki
PhyloPhlAn3 0.30	Asnicar et al.^43^	https://github.com/biobakery/phylophlan
Pyani 0.2.6	Pritchard et al.^44^	https://pypi.org/project/pyani/
Prokka 1.14.6	Seeman,^45^	https://github.com/tseemann/prokka
ROARY 3.13.0	Page et al.^28^	https://github.com/sanger-pathogens/Roary
PRANK v.170427	Löytynoja,^46^	http://wasabiapp.org/software/prank/
Diamond 0.9.9.110	Buchfink et al.^47^	https://github.com/bbuchfink/diamond
MAFFT 7.310	Standley and Katoh,^48^	https://mafft.cbrc.jp/alignment/server/
trimAl 1.2rev59	Capella-Gutiérrez et al.^49^	http://trimal.cgenomics.org/
FastTree 2.1.10	Price et al.^50^	https://bio.tools/fasttree
RAxML 8.1.15	Stamatakis,^51^	https://cme.h-its.org/exelixis/web/software/raxml/
EggNOG mapper 1.0.3	Jensen et al.^52^	https://github.com/eggnogdb/eggnog-mapper
HMMER 3.1b2	Eddy,^53^	http://hmmer.org/
Blast 2.2.31+	Altschul et al.^54^	https://blast.ncbi.nlm.nih.gov/
Bowtie2 2.3.5	Langmead and Salzberg,^55^	http://bowtie-bio.sourceforge.net/bowtie2
SAMtools 1.9	Li et al., 2009, 2011^56^^,^^57^	http://www.htslib.org/
Bcftools 1.9	Danecek et al.2011,2021^58^^,^^59^	https://samtools.github.io/bcftools/bcftools.html
Vcftools 0.1.16	Danecek et al., 2011,2021^58^^,^^59^	http://vcftools.sourceforge.net/
EMBOSS transeq 6.6.0	Rice et al.^60^	https://www.ebi.ac.uk/Tools/st/emboss_transeq/
ClustalW 2.1	Thompson et al.,^61^ Larkin et al.^62^	http://www.clustal.org/clustal2/
PAL2NAL v14	Suyama et al.^63^	https://bio.tools/pal2nal
PAML 4.9j	Yang, 1997, 2007^64^^,^^65^	http://abacus.gene.ucl.ac.uk/software/paml.html

### Resource availability

#### Lead contact

Further information and request for resources and reagents should be directed to and will be fulfilled by the lead contact, Simone Rampelli (simone.rampelli@unibo.it).

#### Materials availability

This study did not generate new unique reagents.

### Experimental model and subject details

#### Human metagenomes

Human metagenome datasets used in this study are from 7 previously published studies, are available in public repositories (see Table S1 for accession numbers), and included 747 subjects spanning different countries (North America, Peru, Sweden, Germany, Italy, Tanzania and Japan) and lifestyles (industrial urban populations, hunter-gatherers and rural communities).

### Method details

#### Constructing a *F. prausnitzii* genome panel with additional curated genomes from metagenomes

A panel of 147 *F. prausnitzii* genomes comprising the entire set of available genomes through the NCBI RefSeq Genome repository (55 genomes, https://www.ncbi.nlm.nih.gov/refseq), and 92 manually curated MAGs (see the paragraph below “metagenomic assembly to MAGs”) were collected for performing the analysis. Metagenomic samples from reference studies^16^^,^^17^^,^^18^^,^^19^^,^^20^^,^^21^^,^^22^ were downloaded via Sequences Read Archive (SRA).^66^ We included gut microbiome samples from individuals from different geographical regions and lifestyles for taking into consideration different aspects of gut microbiome variation. In particular, considered regions were: North America (urbans), Peru (rural inhabitants and hunter-gatherers), Sweden (urbans), Germany (urbans), Italy (urbans), Tanzania (hunter-gatherers) and Japan (urbans). Sequences were quality-checked with FastQC v.0.11.8^35^ and filtered for human reads using KneadData v0.7.2,^36^ in case of single-end reads, and the MetaWRAP command “read_qc” (v1.0.2)^37^ for paired-end reads. The panel was complemented with further 11 genomes from species of the *Ruminococcaceae* family that are considered as outgroup for clade definition in the subsequent analyses. Accession numbers of the *F. prausnitzii* NCBI genomes, metagenomic samples and OS reference genomes included in the study are provided in Table S1.

#### Metagenomic assembly to MAGs

To profile the microbial community composition contained in each quality-filtered sample, shotgun metagenomic sequencing data were analysed with MetaPhlAn2.^38^ Reads from samples containing at least 1% *F. prausnitzii* were assembled using MegaHIT.^39^ The minimum contig length considered for further analyses was set by default to 1kb. MetaBAT 2^40^ and MaxBin 2^41^ algorithms were used for the binning procedure, followed by quality analysis with CheckM.^42^ Only genome bins with >95% bin completeness and <5% bin contamination were retained and taxonomically classified using PhyloPhlAn 3.0^43^ (database*SGB.Dec19*) and MetaWRAP with the NCBI nucleotide and taxonomy databases.^67^ Ninety-two high-quality MAGs classified at species level for *F. prausnitzii* were included within the genome panel.

#### Average nucleotide and genetic distances within the *F. prausnitzii* complex and between the complex and related species

The average nucleotide identity (ANI) pairwise distances were computed using pyani (version 0.2.6; option ‘-m ANIb’)^44^ for all the *F. prausnitzii* genomes and 11 publicly available reference genomes from other species of the *Ruminococcaceae* family included in our panel. Percentage identity was converted into a distance measure, and distances scores were filtered to include only the pairwise comparisons where alignment lengths exceeded 500,000 bp.

The pairwise genetic distances between the same genomes compared above were calculated using a pipeline that included Prokka,^45^ ROARY^28^ and the package “vegan” of the R software.^68^^,^^69^ In brief, each genome was first analysed by Prokka with the ‘--fast’ flag, to identify open reading frames.^70^ The core genome alignments were produced utilizing PRANK^46^ included within the ROARY pipeline. For this step we set the minimum percentage identity for gene clustering to 90% and the minimum required presence for defining core genes to 90% of genomes. The pangenome information obtained, comprising a binary table with gene presence/absence, was used for building a genome-based Jaccard dissimilarity pairwise distance matrix in R using the “vegdist” command.^68^

Clades were finally defined by hierarchical Ward-linkage clustering using both distance matrices. Permutational multivariate analysis of variance was used to verify whether the clades were significantly different from each other in terms of ANI and gene contents (*FDR*< 0.001).

#### Phylogenetic analysis of the *F. prausnitzii* genomes included in the genome panel

A phylogenetic tree was built using the genome panel and PhyloPhlAn 2^26^. The configuration file was customized as by Tett at al.,^71^ using Diamond v0.9.9.110^47^ for the mapping step, MAFFT v7.310^48^ for the multiple sequence alignment, trimAl version 1.2rev59^49^ for trimming, FastTree v2.1.10^50^ for the first tree generated and RAxML v8.1.15^51^ for the final tree. In addition to the customized configuration file, the parameters used were ‘--diversity low --fast’.

#### Identification of clade-specific marker genes and abundance analysis

Marker genes for each clade were identified by analysing the *F. prausnitzii* pangenome obtained with the Prokka and ROARY pipelines (see the “average nucleotide and genetic distances within the F. prausnitzii complex and between the complex and related species” paragraph above for further information). In particular, we defined as “marker genes” for a given clade, the genes present in at least 95% of the genomes of that specific clade and completely absent in all the others (see Table S7 for the number of marker genes identified for each clade). Nucleotide sequences for each pool of marker genes were used for building clade-specific databases with bowtie2-build.^55^ To determine if a given clade was present in a metagenomic sample, the reads were mapped to the clade-specific markers using Bowtie2^55^ and then processed to evaluate the marker genes coverage.^72^ A marker was scored present if it had ≥0.5X coverage and a clade present if at least 50% of its clade-specific markers were hit. Finally, clade relative abundances for each metagenomic sample were calculated as the mean clade marker coverage multiplied by the *F. prausnitzii* genome size (bp) and divided by the metagenome size (bp).

#### Functional annotation

The functional annotation step was performed using the EggNOG mapper (version 1.0.3)^52^ on the protein sequences identified by Prokka with the ‘-d bact’database option. The KEGG Brite Hierarchy was used to screen the EggNOG annotations. Fisher’s exact test with Bonferroni’s correction was used to identify significant differences (p*<*0.01) in gene content between clades.

We also sought for differences in the level of CAZymes.^73^ Gene sequences were identified with HMMSEARCH^53^ against the dbCAN HMMs v6 database,^74^ using default parameters and applying post-processing stringency cut-offs as suggested by the authors (if alignment length >80 AA, E-value is filtered for values < 1e-5, otherwise for values < 1e-3; then a cut-off is applied based on the covered fraction of HMM >0.3).^74^ Only CAZy families that were significantly different in at least one clade (Bonferroni-corrected Fisher’s exact test, p*<*0.01) were retained and graphically represented using the R package “gplots”.^75^

Finally, the genes encoding the MAM protein of *F. prausnitzii* were detected by aligning the protein sequence^31^ against the full set of genes from the *F. prausnitzii* pangenome using protein-protein BLAST (v2.2.31+).^54^ For a complete list of marker genes with annotated function, refer to Table S8.

#### SNP calling procedure and estimation of dN/dS and Tajima’s D values in metagenomic samples

SNP calling procedure was performed for the clade-specific marker genes, 10 selected housekeeping genes (*recA, rplS, rplI, purN, mreB, maf, fmt, gyrB, rpoB, proC*) (Table S3), and 500 randomly selected *F. prausnitzii* core genes as genes present in at least 95% of genomes within our panel. Metagenomic samples showing at least 1% *F. prausnitzii*, ensuing from the previous MetaPhlAn 2^39^ analysis, were aligned against the databases with Bowtie2^55^ using the ‘--end-to-end’ and ‘--very-sensitive’ parameters and then sorted using SAMtools.^56^^,^^57^ Candidate SNPs were identified using BCFtools mpileup,^58^ with the ‘--ploidy’ parameter set to 1, to extract all the variants in vcf format. VCFutils varFilter was then used to filter the minimum depth to 10 reads and the QUAL score >200. For each position, only one point mutation was considered, and the SNP-per-base values were calculated for each gene, dividing the total number of identified SNPs in a gene sequence by its length (bp).

Consensus sequences retrieved from the metagenome alignment and reference sequences were then translated into proteins using EMBOSS transeq 6.6.0^60^ and the proteins were aligned using ClustalW 2.1.^61^^,^^62^ Protein alignment was converted into codon-aligned PAML alignment using PAL2NAL v14^63^ and analyzed using the CODEML program of the PAML phylogenetic analysis package (v4.9j),^64^^,^^65^ to compute dN/dS. Codon frequencies were set to ‘3 × 4’ and no phylogenetic tree was submitted. The outputs of the pairwise comparison between reconstructed consensus genes from metagenomes and reference genes were considered and filtered for 0.01 < dS < 2, because values of dS ≤ 0.01 or ≥ 2 entail unreliable estimate of dN/dS since the sequences are too similar or too divergent.

Tajima’s D values were computed with vcftools 0.1.16^59^ over each gene sequence starting from previously identified and quality-filtered polymorphisms. Both population genetic parameters (dN/dS and Tajima’s D) for the *F. prausnitzii* clades were calculated for the same set of marker genes and 500 core genes used for the SNP calling procedure. The parameters were calculated separately for each gene, then the median values were used to represent the parameters for each specific clade.

#### Implementation of divergence rate indices (DRIs) and Non-synonymous divergence rate indices (NDRIs)

In this study we introduced Divergence Rate Indices (DRIs) and Non-Synonymous Divergence Rate Indices (NDRIs), as clade- or gene-specific indices to assess sequence divergence.

DRI indices were estimated using the SNP-per-base values previously computed. For each metagenomic sample we calculated the DRI for a specific gene of interest (DRIg), using the number of SNP-per-base detected for that specific gene of interest (M_G_), the median number of SNP-per-base detected for 10 housekeeping genes (M_H_), and calculating the ln of the ratio between the two values. Analogously, we defined the clade-level DRI (DRIc) by considering the median number of SNP-per-base for the entire set of clade-specific genes (M_E_), the median value of SNP-per-base for the set of housekeeping genes (M_H_) and calculating the ln of the ratio between the two values.

On the other hand, NDRI indices were estimated using the dN/dS values previously computed. For each metagenomic sample we calculated the NDRI for a specific gene of interest (NDRIg), using the value of dN/dS ratio detected for that specific gene of interest (μ_G_), the mean value of dN/dS ratio detected for the 10 housekeeping genes (μ_H_), and calculating the ln of the ratio between the two values. Analogously, we defined the clade-level NDRI (NDRIc) by considering the mean value of dN/dS ratio for the entire set of clade-specific genes (μ_E_), the mean value of dN/dS ratio for the set of housekeeping genes (μ_H_) and calculating the ln of the ratio between the two obtained values.

$$\mathrm{DRIc}=\;\ln\frac{M_{E}}{M_{H}}\;\mathrm{DRIg}=\;\ln\frac{M_{G}}{M_{H}}$$

$$\mathrm{NDRIc}=\ln\frac{\mu_{E}}{\mu_{H}}\;\mathrm{NDRIg}=\;\ln\frac{\mu_{G}}{\mu_{H}}$$

Higher values for all indices indicate a higher number of SNPs or non-synonymous substitutions in the specific gene/group of genes compared to housekeeping genes.

When M_H_ or μ_H_ values were equal to zero, we substituted the value with the lowest M_H_ or μ_H_ detected in the global human population. Furthermore, when both dividend and divisor were equal to zero, we set the indices to zero. These corrections had no effect on our results since we only focused on positive values to determine the divergence.

### Quantification and statistical analysis

Statistical analysis was performed using R software v4.0.3. The pairwise genetic distances between the same genomes of the panel obtained via the Prokka/ROARY pipeline were analysed with the R package “vegan”. The Jaccard dissimilarity pairwise distance matrix was built using the “vegdist” command. Permutational multivariate analysis of variance was used to verify whether the clades were significantly different from each other in terms of ANI and gene contents (p value corrected for multiple testing applying Benjamini-Hochberg false discovery rate,^76^*FDR*< 0.001). Fisher’s exact test with Bonferroni’s correction was used to identify significant differences (p< 0.01) in gene content and CAZymes counts between clades. Graphical representations were made using the R packages “gplots”, “ggplot2”.

## Data Availability

• All human gut metagenomic sequences used in this study are available in public repositories (see Table S1 for accession numbers). • This paper does not report original code. • Any additional information required to reanalyze the data reported in this paper is available from the lead contact upon request

## Supplemental information

Document S1. Figures S1–S4 and Tables S2, S3, S6 and S7

Table S1. Accession number of NCBI genomes of *F. prausnitzii*, other species of the *Ruminococcaceae* family used for the analysis, and metagenomic samples included for the starting dataset, related to Figure 1 For each metagenomic sample, the accession number and the BioProject code is reported. For each *F. prausnitzii* reference genome downloaded from the NCBI database, information concerning strain identification, level of genome assembly, genome size (Mb), percentage of GC content, the number of scaffold, the release date of the sequence and the correspondent RefSeq FTP link are listed. Reference genomes for the OS are given with the assembly code, the considered strain, as well as the BioSample and BioProject codes

Table S4. Gene-level divergence values (DRIg and NDRIg) of the marker genes, related to Figure 3 For each marker gene, the two computed values of our indices are reported, together with the clade belonging, and the identified gene name and KO when available

Table S5. List of KO functionalities under diverging pressure and their presence (1) or absence (0) in the 5 *F. prausnitzii* clades, related to Figure 5

Table S8. Putative functions of the identified *F. prausnitzii* clades-specific marker genes, related to Figure 3 For each clade-specific marker gene the clade, gene name assignment, KO and EggNOG putative function description are reported
