# Supplementary material for: Exploring clade differentiation of the Faecalibacterium prausnitzii complex
Source: iScience. 2022 Nov 9;25(12):105533. doi: 10.1016/j.isci.2022.105533 (PMC9676633; doi:10.1016/j.isci.2022.105533)
Supplement: Document S1. Figures S1–S4 and Tables S2, S3, S6 and S7 [file mmc1.pdf]

iScience, Volume *25*

Supplemental information

Exploring clade differentiation of the *Faecalibacterium prausnitzii* complex

Marco Fabbrini, Marco Candela, Silvia Turroni, Patrizia Brigidi, and Simone Rampelli

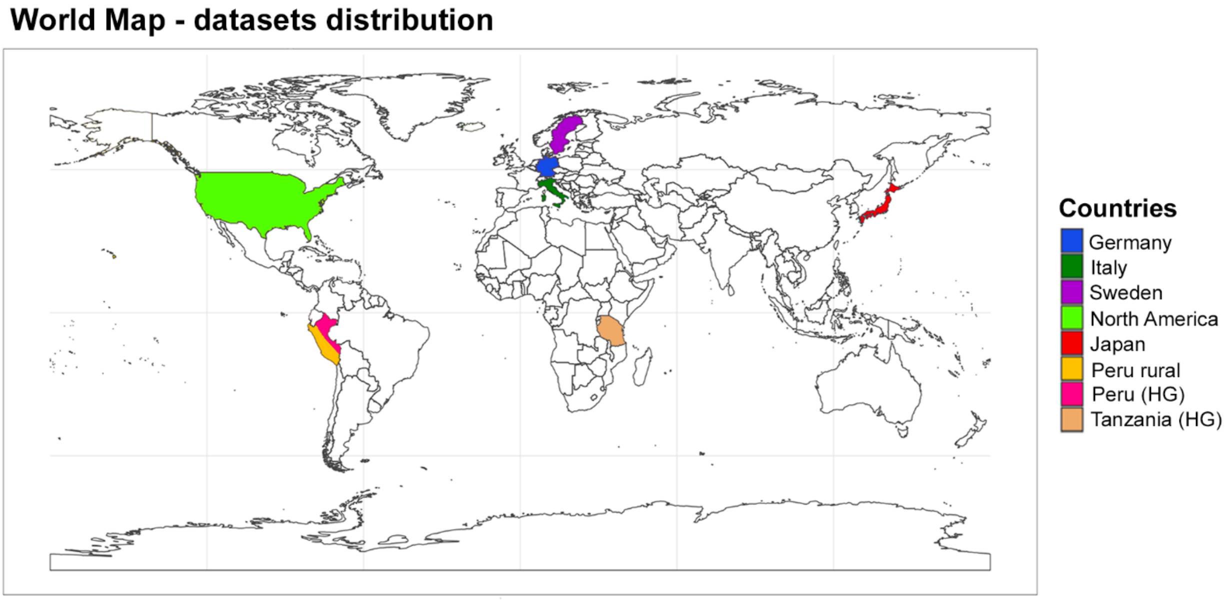

Supplementary Figure 1. Global distribution of metagenomic samples, related to Figure 1.
World map representing the distribution of the 747 metagenomic samples analyzed in this work. HG, hunter-gatherers.

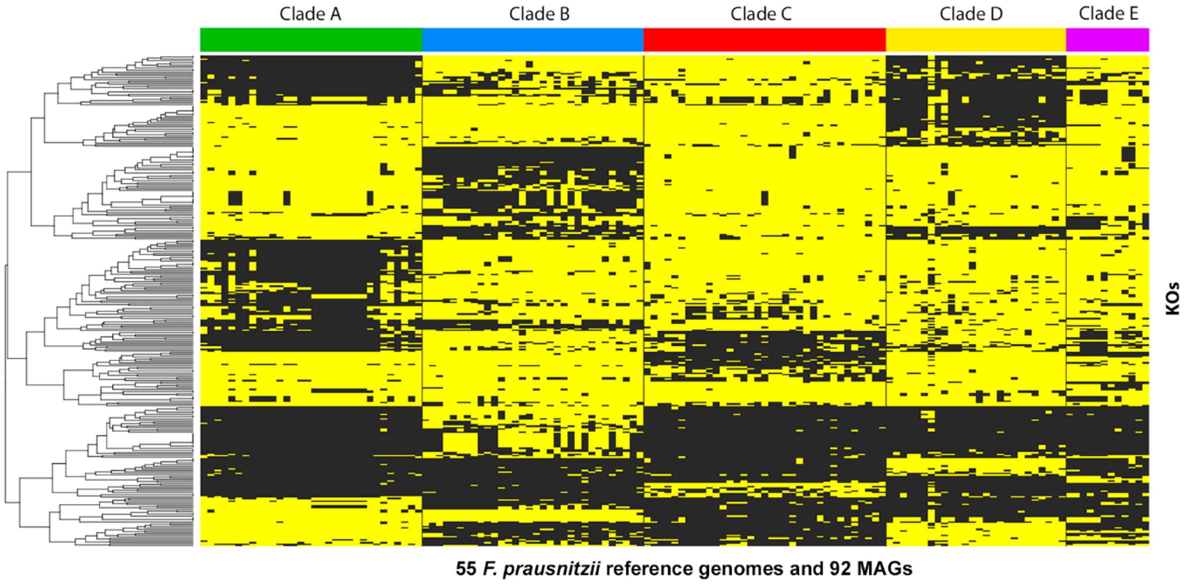

Supplementary Figure 2. The 5 identified clades of the *F. prausnitzii* complex showed different functionality, related to Figure 1.
Presence (yellow) and absence (black) of KEGG KOs differently represented between the genomes and MAGs of the 5 *F. prausnitzii* clades (A to E). Only KOs with $p < 0.01$ (Bonferroni-corrected Fisher's exact test) are shown.

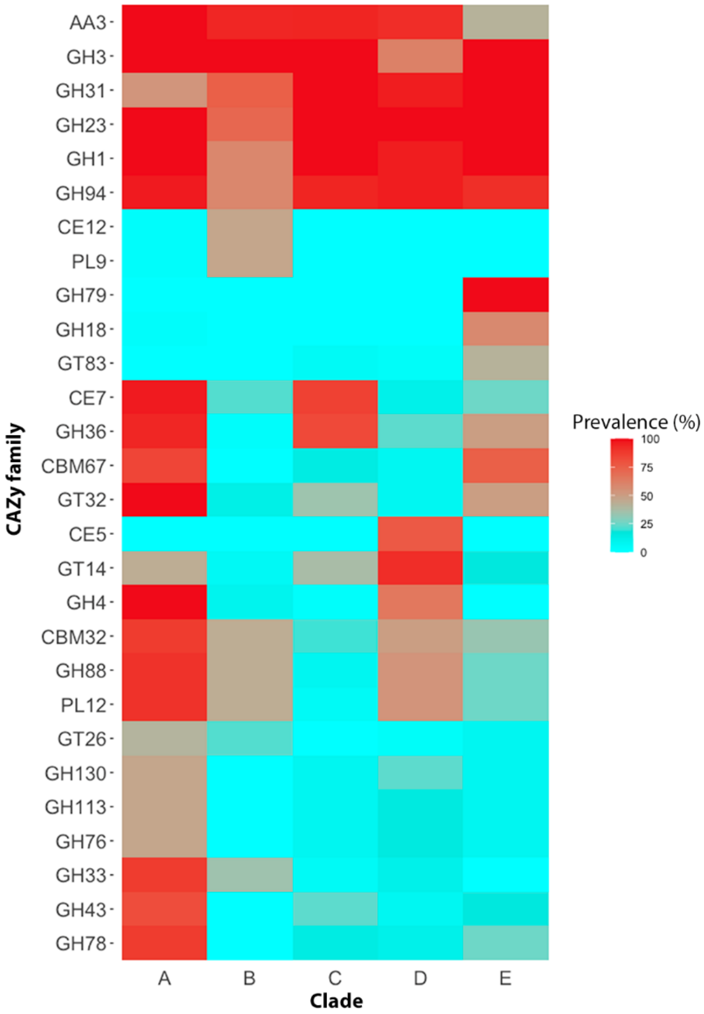

Supplementary Figure 3. The 5 *F. prausnitzii* clades show a different carbohydrate degradative potential, related to Figure 1.

CAZy families significantly (Bonferroni-corrected Fisher's exact test, $p < 0.01$) enriched or depleted in at least one *F. prausnitzii* clade (A to E) are shown. Prevalence is defined as the percentage of genomes in a clade for which at least one copy of a gene belongs to the given CAZy family. AA, auxiliary activity; GH, glycoside hydrolase; CE, carbohydrate esterase; PL, polysaccharide lyase; GT, glycosiltransferase; CBM, carbohydrate-binding module.

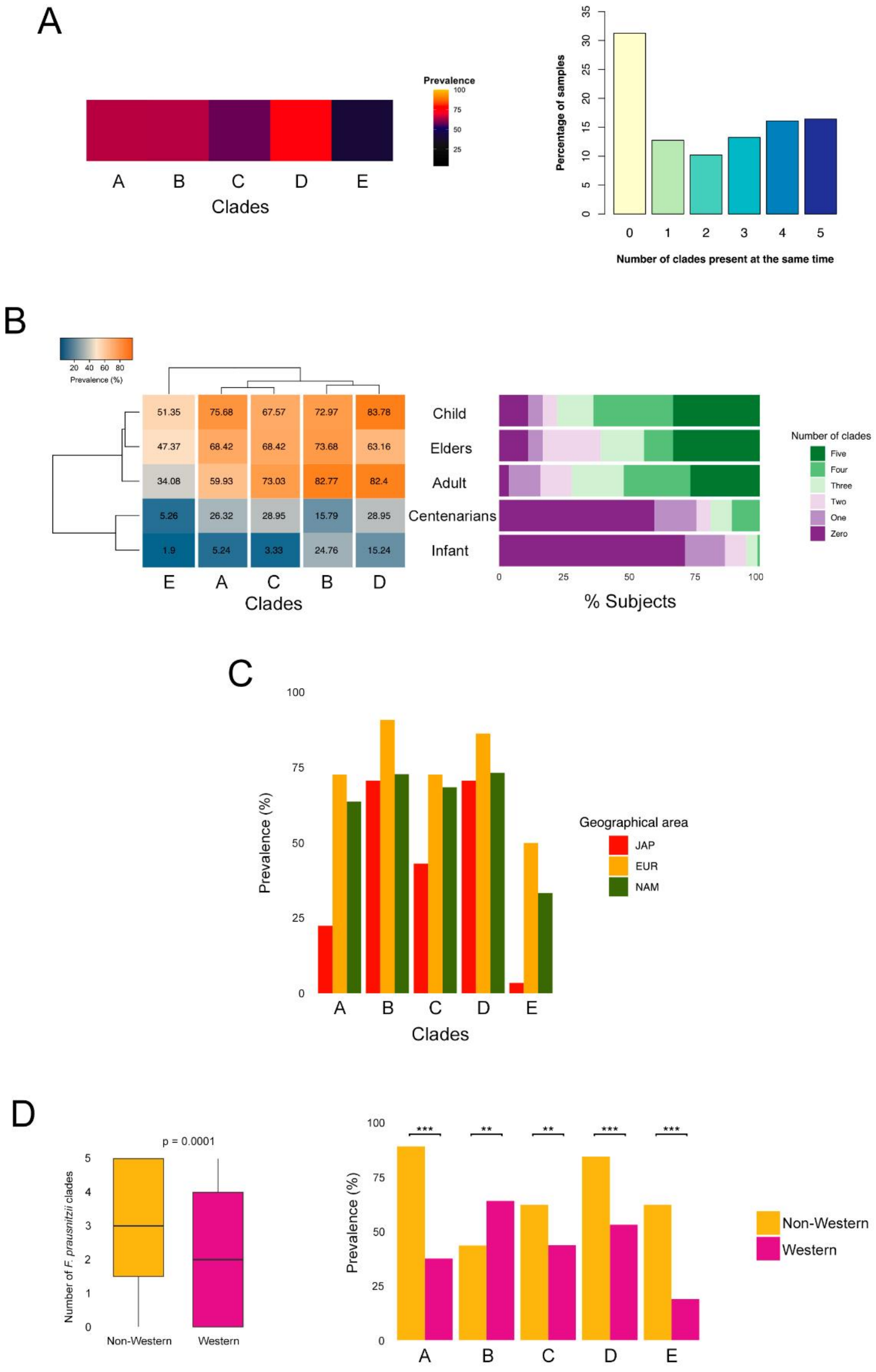

Supplementary Figure 4. Differences in *F. prausnitzii* clades prevalence and copresence in the human population, related to Figure 1.

(**A**) The 5 identified clades (A to E) of the *F. prausnitzii* complex showed significantly different prevalence across the 747 metagenome samples analyzed in this study. Looking at the copresence (right graph) of multiple clades within an individual, we confirmed that multiple clades can be hosted in the same subject. Two hundred and thirty-three individuals did not carry *F. prausnitzii* at all, whilst the copresence of one to five clades showed comparable values.

(**B**) The five clades were differently distributed in the human population according to age, with infant and centenarians showing the lowest prevalence. The presence of at least one clade was scores

(graph on the right) in 28.7% of the infant metagenomes and in 40.5% of the centenarian ones. 88.8%of individuals for both Child and Elders carried at least one of the five clades. Adults showed the higher percentage of individuals carrying at least one clade (96.2% of them). Infant < 1 years old, 1-15 Child, 18-69 Adult, 70-97 Elders, >99 Centenarians

(**C**) Prevalence of the different *F. prausnitzii* clades in adults grouped by geographical location (JAP, Japan; EUR, Europe; NAM, North America).

(**D**) Number of co-occurring clades in the same subject in Western and Non-Western population (p value was computed using Wilcoxon's test,), and prevalence (graph on the right) of the different *F. prausnitzii* clades in subjects grouped by subsistence strategies (p values were computed using Fisher's test. ***p < 0.001, **p<0.01).

The five clades identified in this work	De Filippis et al., 2020
**A**	B, G
**B**	A
**C**	C
**D**	D, K, J
**E**	E, F, H, I

Supplementary Table 2. Comparison of the 5 *F. prausnitzii* clades identified within this work with the 11 previously defined by De Filippis et al., 2020, related to Figure 1.

Gene	Role	Function (UniProt)
recA	Repair and maintenance of DNA	Required for homologous recombination and the bypass of mutagenic DNA lesions by the SOS response. Catalyzes ATP-driven homologous pairing and strand exchange of DNA molecules necessary for DNA recombinational repair. Catalyzes the hydrolysis of ATP in the presence of single-stranded DNA, the ATP-dependent uptake of single-stranded DNA by duplex DNA, and the ATP-dependent hybridization of homologous single-stranded DNAs. The SOS response controls an apoptotic-like death (ALD) induced (in the absence of the mazE-mazF toxin-antitoxin module) in response to DNA damaging agents that is mediated by RecA and LexA.
rplS	50S ribosomal protein	This protein is located at the 30S-50S ribosomal subunit interface. In the 70S ribosome it has been modeled to make two contacts with the 16S rRNA of the 30S subunit forming part of bridges B6 and B8.
rplI	50S ribosomal protein	One of the primary rRNA binding proteins, it binds very close to the 3' end of the 23S rRNA.
purN	Phosphoribosylglycinamide formyltransferase	Catalyzes the transfer of a formyl group from 10-formyltetrahydrofolate to 5-phospho-ribosyl-glycinamide (GAR), producing 5-phospho-ribosyl-N-formylglycinamide (FGAR) and tetrahydrofolate.
mreB	Cell-shape determining protein	Forms membrane-associated dynamic filaments that are essential for cell shape determination (PubMed:15612918, PubMed:21903929). Acts by regulating cell wall synthesis and cell elongation, and thus cell shape (PubMed:21903929). A feedback loop between cell geometry and MreB localization maintains elongated cell shape by targeting cell wall growth to regions of negative cell wall curvature (PubMed:24550515). Filaments rotate around the cell circumference in concert with the cell wall synthesis enzymes. The process is driven by the cell wall synthesis machinery and does not depend on MreB polymerization (PubMed:21903929). Rotation may contribute to the robust maintenance of rod shape (PubMed:21903929).
maf	Nucleotide binding protein	Nucleoside triphosphate pyrophosphatase that hydrolyzes dTTP and UTP. May have a dual role in cell division arrest and in preventing the incorporation of modified nucleotides into cellular nucleic acids.
fmt	tRNA modification	Attaches a formyl group to the free amino group of methionyl-tRNA(fMet). The formyl group appears to play a dual role in the initiator identity of N-formylmethionyl-tRNA by promoting its recognition by IF2 and preventing the misappropriation of this tRNA by the elongation apparatus.
gyrB	DNA topology	A type II topoisomerase that negatively supercoils closed circular double-stranded (ds) DNA in an ATP-dependent manner to modulate DNA topology and maintain chromosomes in an underwound state. Negative supercoiling favors strand separation, and DNA replication, transcription, recombination and repair, all of which involve strand separation. Also able to catalyze the interconversion of other topological isomers of dsDNA rings, including catenanes and knotted rings. Type II topoisomerases break and join 2 DNA strands simultaneously in an ATP-dependent manner.
rpoB	DNA-dependent RNA polymerase	DNA-dependent RNA polymerase catalyzes the transcription of DNA into RNA using the four ribonucleoside triphosphates as substrates
proC	Pyrroline-5-carboxylate reductase	Catalyzes the reduction of 1-pyrroline-5-carboxylate (PCA) to L-proline

Supplementary Table 3. Housekeeping genes list considered in this study and their function from UniProt database, related to Figure 2.

UNIQUE FUNCTIONS - CLADE A	COUNTS
Biosynthesis of cofactors	7
Sporulation protein	6
DNA repair	4
Nucleotide sugar metabolism	4
Transcription factor	3
Transporter	3
Microbial metabolism in diverse environments	3
Terpenoid backbone biosynthesis	2
Antibiotic biosynthesis	2
Glycerolipid metabolism	2
Nicotinate and nicotinamide metabolism	2
Ribosome biogenesys	2
Two-component system	1
Xenobiotic degradation	1
CRISPR protein	1
Antimicrobial resistance	1
tRNA biogenesis	1
Aminoacyl-tRNA biosynthesis	1
Transcription machinery	1
Peptidoglycan biosynthesis	1
Purine metabolism	1
Lipopolysaccharide biosynthesis	1
Peptide deformylase	1
Glycine, serine and threhonine metabolism	1
Hydrolase	1
Phosphohydroalse	1
Protein export	1
Dehydrogenase	1
Cell division protein	1
Biotin metabolism	1
Phenylalanine, tyrosine and tryptophan biosynthesis	1
Phospholipase	1
Peptidase	1
Tat system	1
Fructose and mannose metabolism	1
Phosphatase	1

UNIQUE FUNCTIONS - CLADE B	COUNTS
Two-component system	7
Antibiotic resistance	6
Amino acid transport and metabolism	5
Transporter	5
Peptidoglycan biosynthesis	3
Carbohydrate transport and metabolism	2
Nucleotide repair	1
Sulfur relay system	1
Sporulation	1
Ribosome biogenesis	1
tRNA biogenesis	1
Biosynthesis of cofactors	1
Replication	1
Glycerolipid metabolism	1
Phosphatase	1
Nitrogen metabolism	1
Oleic acid detoxification	1
Glycosyltransferase	1
Starch and sucrose metabolism	1
Flagellar assembly	1
Nicotinate and nicotinamide metabolism	1

UNIQUE FUNCTIONS - CLADE C	COUNTS
Efflux system	5
Biosynthesis of cofactors	4
Antibiotic resistance	4
Amino acid transport and metabolism	3
Sporulation	3
tRNA biogenesis	2
Transcription factor	2
Microbial metabolism in diverse environment	2
DNA repair	2
Cell division	2
Signal transduction	2
mRNA metabolism	1
Sulfur-relay system	1
Xenobiotic degradation	1
Ribosome biogenesis	1
Transcription machinery	1
Protein export	1
Transporter	1
One carbon pool	1

UNIQUE FUNCTIONS - CLADE D	COUNTS
Inorganic ion transporter	4
Machanosensitive channel	1
Niacin transporter	1
Selenocompound metabolism	1
Peptidoglycan biosynthesis	1
Lipid transport and metabolism	1
Amino acid transport and metabolism	1
Biosynthesis of cofactors	1

UNIQUE FUNCTIONS - CLADE E	COUNTS
Amino acid transport and metabolism	2
Prokaryotic cell cycle	2
Starch metabolism	2
Xenobiotic degradation	1
Chaperone	1
Lipid transport metabolism	1
Carbohydrate transport and metabolism	1
Energy production and conversion	1

Supplementary Tables 6. KOs of diverging functionalities identified for each *F. prausnitzii* clade, related to Figure 3.
KOs corresponding to the most diverging clade-specific genes identified by selecting those with DRIg > 0 and NDRIg > 0.

Clade	N° of clade-specific marker genes
A	565
B	381
C	496
D	178
E	122

Supplementary Table 7. Number of clade-specific marker genes identified for each *F. prausnitzii* clade, related to Figure 3.
